# Supplementary material for: The Cytokine Nicotinamide Phosphoribosyltransferase (eNAMPT; PBEF; Visfatin) Acts as a Natural Antagonist of C-C Chemokine Receptor Type 5 (CCR5)
Source: Cells. 2020 Feb 21;9(2):496. doi: 10.3390/cells9020496 (PMC7072806; doi:10.3390/cells9020496)
Supplement: Supplementary file 1 [file cells-09-00496-s001.pdf]

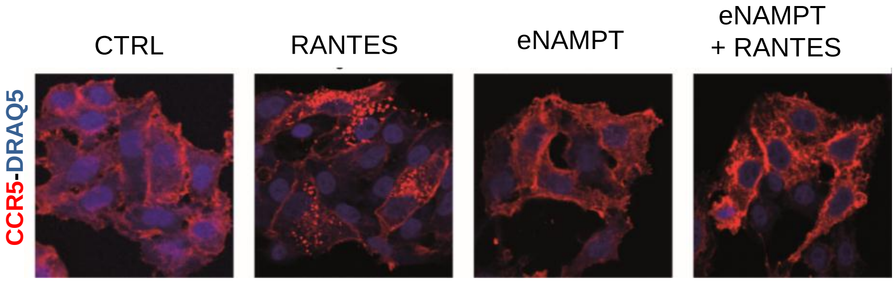

Supplementary Figure S1. Immunofluorescence of CCR5 localization in Hela-CCR5: cells were treated with vehicle, Rantes (100 ng/ml = 9 nM) and/or eNAMPT (2.5 µg/ml = 45 nM) for 60 minutes at 37°C or 4°C. Then, cells were fixed in 4% formaldehyde and stained with an anti-mouse CCR5-PE and DRAQ5 (nuclear staining).

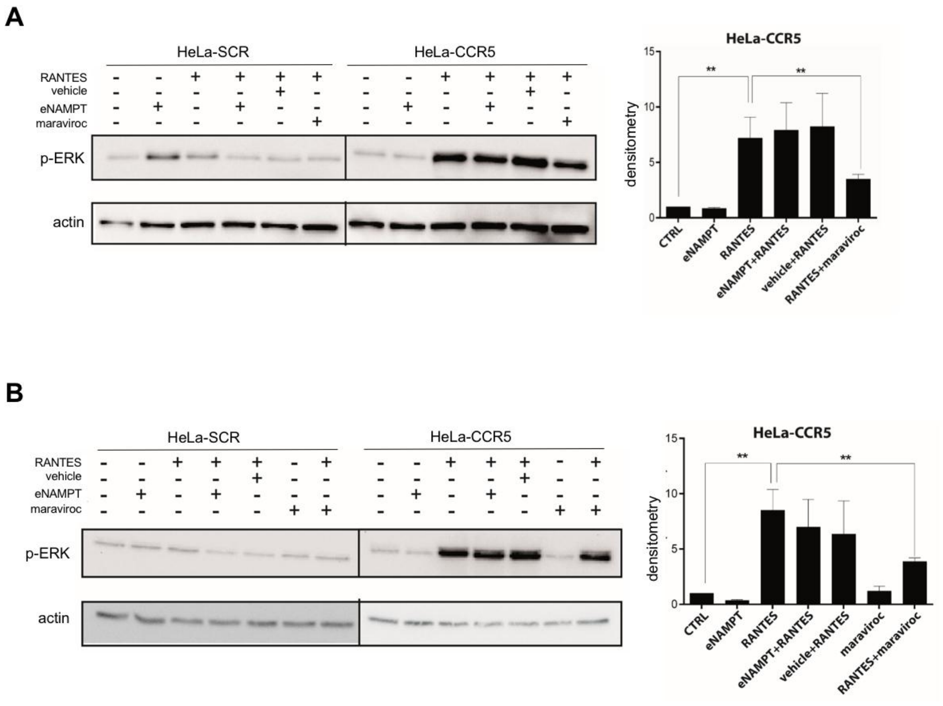

Supplementary Figure S2. (**A**) Representative Western blot and densitometry analysis of phosphorylated ERK after 2 hours of starvation followed by co-treatment for 5 minutes with recombinant RANTES (25 ng/mL = 3 nM) in the presence of eNAMPT (500 ng/mL = 9 nM) or maraviroc (10 µM) or vehicle in serum-free conditions. Data from 4 separate experiments. (**B**) Representative Western blot and densitometry analysis of phosphorylated ERK after 2 hours of starvation followed by pre-treatment for 45 minutes with recombinant eNAMPT (500 ng/mL = 9 nM) or maraviroc (10 µM) or vehicle followed by treatment for 5 minutes with RANTES (25 ng/mL = 3 nM) in serum-free conditions. Data from 3 separate experiments.

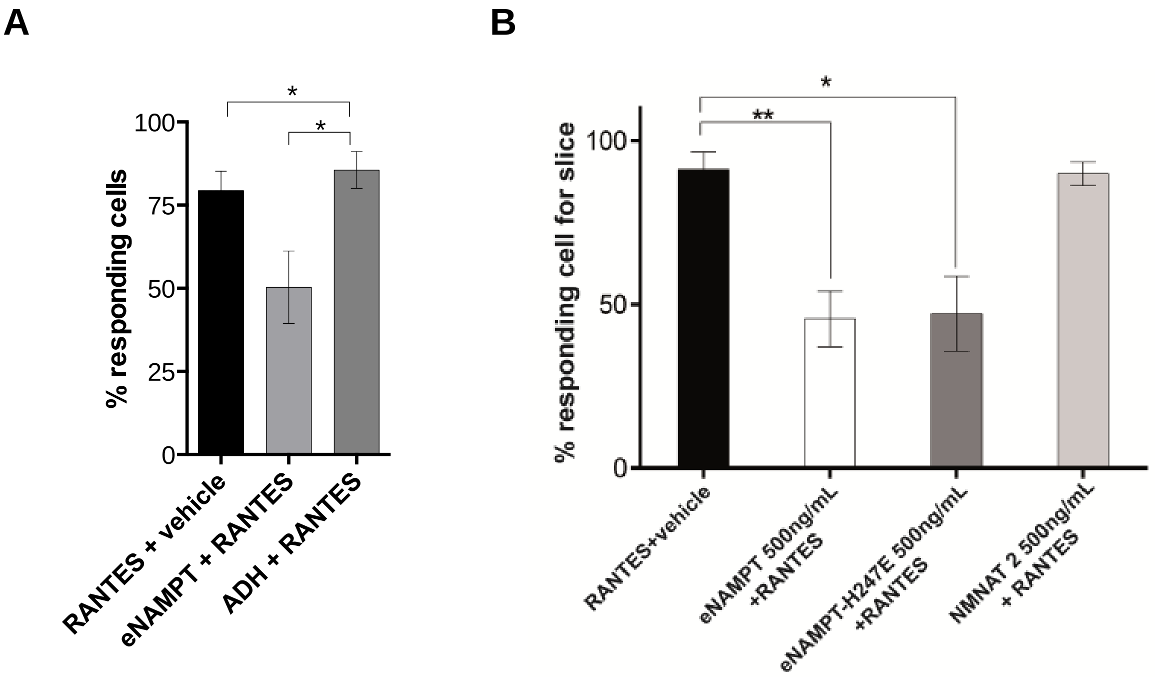

Supplementary Figure S3 (**A**) Calcium release in HeLa-CCR5 loaded with FURA-2AM and treated with vehicle, eNAMPT (500 ng/ml) or ADH (500 ng/mL) or for 100 sec before the addition of 25 ng/ml of Rantes. Histograms of responding cells. (**B**) Calcium release in HeLa-CCR5 loaded with FURA-2AM and treated with vehicle, eNAMPT (500 ng/ml), H247E eNAMPT (500 ng/mL) or NMNAT2 (500 ng/mL) for 100 sec before the addition of 25 ng/ml of Rantes. Histograms of responding cells.

A

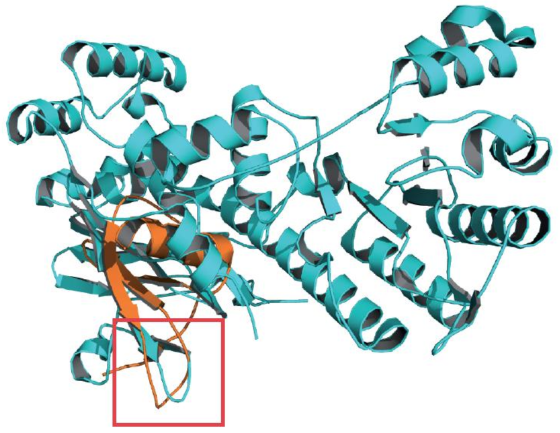

B

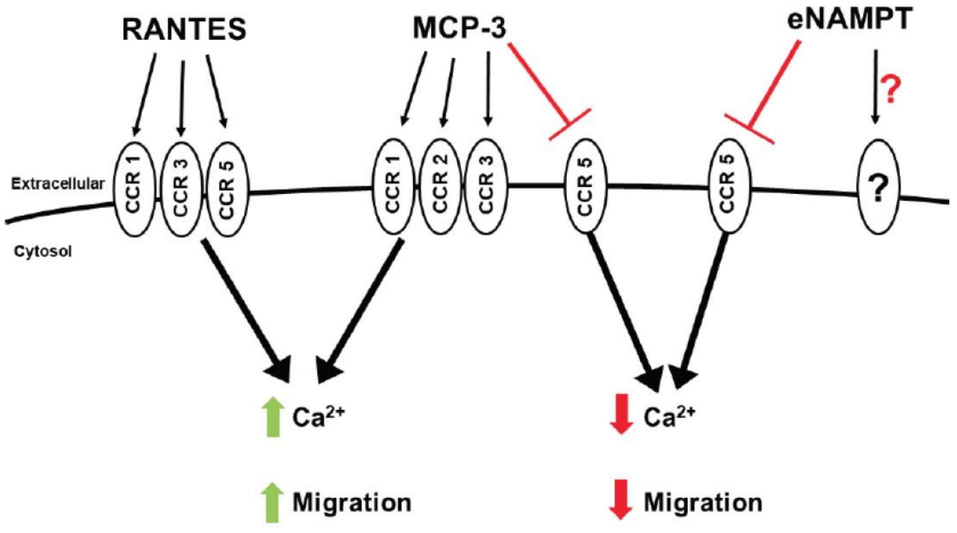

Supplementary Figure S4. (**A**) PyMol and Uniprot analysis of NAMPT (cyano) and CCL7 (orange). CCL7 random coil, which is used by the protein to bind to CCR5, is highlighted by the red square and is superimposable with a portion of NAMPT structure (amino acids 420-430). (**B**) Scheme of Rantes, CCL7 and eNAMPT signalling.
